# Comparative analysis of Sarcopenia in hospitalized elderly: exploring the impact of liver cirrhosis

**DOI:** 10.1007/s11739-024-03709-1

**Published:** 2024-07-19

**Authors:** A. D. Romano, M. G. Cornacchia, M. Sangineto, G. Di Gioia, R. Villani, G. Serviddio

**Affiliations:** Internal Medicine and Liver Unit, Department of Medical and Surgical Sciences, University of Foggia, Policlinico Riuniti, 71122 Foggia, Italy

**Keywords:** Sarcopenia, Appendicular Skeletal Muscle Mass (ASM), Liver, Cirrhotic, Mini Nutritional Assessment (MNA)

## Abstract

The progressive aging of the population has led to a rise in geriatric pathologies, with sarcopenia, characterized by muscle mass and function loss, becoming a crucial prognostic indicator. This study investigates sarcopenia in elderly hospitalized patients with advanced chronic liver disease (cirrhotic) and non-liver disease patients, comparing their prevalence and exploring correlations with anthropometric and biochemical factors. The cohort of 115 patients, including 50 cirrhotic and 65 non-cirrhotic individuals, exhibited significant comorbidities and a mean age of 78.4 years. Cirrhotic patients presented distinct laboratory parameters indicating liver damage. Applying European Working Group on Sarcopenia in Older People criteria, probable sarcopenia prevalence was similar in cirrhotic (62%) and non-cirrhotic (63%) patients. Stratifying probable sarcopenia into confirmed sarcopenia and dynapenia revealed no significant differences between populations. Correlation analyses demonstrated positive associations between Appendicular Skeletal Muscle Mass (ASM) and anthropometric parameters, malnutrition risk, and grip strength. In cirrhotic patients, muscle mass inversely correlated with liver damage. Odds ratio analysis highlighted the Mini Nutritional Assesment’s (MNA) significant predictive capability for sarcopenia. ROC curve analysis affirmed MNA and biochemical markers' combined use, such as transferrin, albumin, total cholesterol, lymphocyte count and C-reactive protein as a strong predictor. Despite limitations, such as a small sample size, this study underscores the significance of thorough sarcopenia screening in elderly hospitalized patients, especially those with cirrhosis. Indeed, individuals with end-stage liver disease are particularly susceptible to sarcopenia. A more personalized approach utilizing tools like MNA and biochemical markers could prove beneficial. Further research is warranted to validate these findings and inform clinical interventions.

## Introduction

The progressive aging of the population has given rise to an increase in geriatric pathologies. Sarcopenia, a geriatric condition characterized by a gradual loss of muscle mass and function, origin from the Greek words “sarx” (“σάρξ”) and “penia” (“πενία”), meaning “muscle” and “poverty,” respectively. It is considered a crucial prognostic indicator for survival and the risk of complications, particularly in specific populations such as patients with liver cirrhosis and metabolic diseases [1, 2]. Traditionally associated with aging, recent studies have revealed its early onset in adulthood and the existence of various phenotypes caused by etiological factors beyond aging. It is classified into: “primary” (age-related) when no other specific cause is evident, “secondary” when multiple causal factors, such as systemic inflammatory diseases, cancer, organ failure, physical inactivity and malnutrition, contribute. Identifying sarcopenia is crucial for initiating early clinical interventions and formulating strategies to prevent or postpone its progression to frailty syndrome [3], particularly considering the technical difficulties associated with precise measurements of both muscle mass and quality. With improving life expectancy, the global population aged over 60 continues to rise, inevitably leading to an increased prevalence of sarcopenia. In certain populations, such as patients with diabetes mellitus [1] (18%) and those with unresectable esophageal cancer [4] (up to 66%), sarcopenia's prevalence exceeds that of the general elderly population [5]. Elevated prevalence rates are also observed in patients with chronic kidney disease [6] (25.6%) and advanced chronic liver disease [2, 7](37.50%), as well as in patients with tumors in various districts [8].

Liver cirrhosis represents the ultimate evolution of chronic liver disease, irrespective of its etiology. Metabolically, it induces significant alterations in all major biochemical-nutritional patterns. Among the observable metabolic changes, protein metabolism is particularly affected, marked by a predominant catabolic state wherein the degradation of muscle proteins exceeds synthesis, leading to the development of sarcopenia [9]. In cirrhotic patients, sarcopenia prevalence ranges from 30 to 70%, depending on the underlying etiology and assessment modalities. It is more prevalent in male patients and those with Child–Pugh class C functional status [10, 11]. Alcoholic steatohepatitis (ASH) remains a common etiology associated with sarcopenia, yet non-alcoholic steatohepatitis (NASH) is on the rise due to the obesity and diabetes epidemic [12, 13]. Treatment strategies for ASH and NASH, such as reduced caloric intake and weight loss, may exacerbate underlying sarcopenia.

Multiple mechanisms contribute to sarcopenia in cirrhotic patients, including malnutrition, altered skeletal muscle protein synthesis, iatrogenic factors, and systemic inflammation [9, 14, 15]. Sarcopenia in cirrhotic patients is associated with increased complications (e.g., hepatic encephalopathy and infections) and mortality [16]. The nutritional aspect plays a pivotal role in managing cirrhotic patients, with malnutrition defined as a state resulting from insufficient nutrient intake or absorption, leading to alterations in body composition and function, associated with adverse outcomes in response to disease [17–19]. Malnutrition associated with inflammation, identified by a C-reactive protein (CRP) level > 5 mg/L, is recognized as a syndrome linked to an underlying disease, characterized by muscle mass loss with or without fat mass loss [20].

Assessment of sarcopenia in advanced chronic liver disease involves techniques such as cross-sectional skeletal muscle imaging at the L3 vertebra level using computed tomography (CT). This method correlates significantly with overall body muscle mass and serves as a predictor of mortality [21]. While CT usage is limited in clinical practice due to cost and radiation exposure, it can be employed during screenings for hepatocellular carcinoma or liver transplantation evaluations, study of vascular shunts and/or portal thrombosis and it can also be used collaterally for the assessment of sarcopenia [19]. Other assessment methods, including dual-energy X-ray absorptiometry (DEXA), bioelectrical impedance analysis (BIA), and skeletal muscle strength evaluation, offer alternative approaches with their unique advantages and limitations such as fluid retention that is often present in cirrhotic patients [22].

In accordance with the operational definition of sarcopenia from 2018 [23], probable sarcopenia is recognized through low muscle strength, while diagnosis is confirmed by documenting low muscle quantity or quality. Our primary endpoint was to assess sarcopenia in elderly hospitalized patients with advanced chronic liver disease and compare it with that of non-hepatopathic patients, while also examining potential correlations with anthropometric and biochemical factors. More in dept, we determined the prevalence of “probable sarcopenia” and, through the approximation of Appendicular Skeletal Muscle Mass (ASM), “confirmed sarcopenia” in the two populations of elderly hospitalized patients, distinguishing it from “dynapenia.” In addition, we will differentiate between patients with confirmed sarcopenia and those without confirmation, examining the differences within these subgroups. Our secondary endpoint was to identify a diagnostic test capable of predicting the risk of sarcopenia in the elderly, particularly in a population at high risk of malnutrition such as cirrhotic patients. Employing biochemical parameters related to malnutrition, inflammation, and malnutrition screening tests (MNA), we assessed the reliability of these tests in predicting sarcopenia in both cirrhotic and non-hepatopathic patient subgroups. This comprehensive analysis aims to enhance our understanding of sarcopenia in elderly hospitalized individuals with advanced chronic liver disease and its association with various clinical and biochemical factors.

## Material and methods

We conducted a comprehensive cross-sectional observational study at the Hepatology Unit of the Polyclinic of Foggia (FG) and the Geriatrics Unit of the Hospital “Miulli” in Acquaviva delle Fonti (BA), Italy. We designed it focusing on collecting data at a single point in time to examine relationships or characteristics within the population at that specific moment. Prior to participation, all individuals provided written informed consent, and the study was approved by the institutional ethics committee. Our research focused on a cohort of 115 patients consecutively admitted in each ward during the period spanning from April 2022 to July 2023. All of whom were enlisted to provide a comprehensive understanding of sarcopenia in elderly individuals with and without advanced chronic liver disease. To be eligible for inclusion in the study, participants had to be aged over 65 years, while exclusion criteria encluded the absence of informed consent, fractures of the upper and lower limbs, paresis, amputations, pre-existing bed rest syndrome, advanced cognitive impairment, and hepatic encephalopathy. Since our goal was to verify whether sarcopenia in cirrhotic patients differed from that in non-cirrhotic patients, this approach aimed to minimize selection bias and provide a comprehensive understanding of sarcopenia in this patient population.

Upon admission, a series of assessments and tests were conducted to gather pertinent data. Venous sampling for peripheral routine blood tests was performed, providing baseline information about the patients’ health status. Furthermore, detailed anamnestic data on the patients’ pathologies and the etiology of cirrhosis were collected, offering valuable insights into the context of each case.

Muscle strength was assessed using the Hand Grip, employing a calibrated handheld dynamometer (digital grip strength dynamometer Chorder, model MG4800). We conducted measurements following the guidelines outlined in the Southampton protocol for assessing adult grip strength. Participants were seated comfortably with their forearms resting on the chair arms. We administered three grip strength tests for each hand, recording the highest measurement for analysis. A minimum rest period of 30 s was allowed between each measurement. Grip strength was defined as the maximum recorded measurement from the dominant hand [24]. To gauge body composition, the Body Mass Index (BMI) was determined by measuring weight and height. Additional anthropometric measurements included arm circumference (mid upper arm circumference) and calf circumference, providing a more comprehensive understanding of the patients’ physical status.

Nutritional assessments were conducted using the Mini Nutritional Assessment (MNA) questionnaire. According to Cruz-Jentoft et al., patients were classified as having “probable sarcopenic” or “sarcopenic”" based on evidence of low muscle strength (with handgrip strength -HGS below 27 kg in males and 16 kg in females) and, when applicable, the presence of reduced muscle mass, calculated using the appendicular skeletal muscle mass (ASM) estimate formula, using the equation proposed by Hwang et al. (cut offs are ASM < 20 kg in men and < 15 kg in women) [23, 25]. Moreover, individuals diagnosed with “dynapenia” are characterized by low muscle strength, using the same handgrip strength (HGS) criteria as employed for sarcopenia, while concurrently exhibiting normal calculated appendicular skeletal muscle mass (ASM) values (> 20 kg in males and > 15 kg in females) [23]. To further assess nutritional status, various laboratory parameters associated with malnutrition were considered, such as transferrin < 200 mg/dl, albumin < 3.5 g/dl, total cholesterol < 160 mg/dl, lymphocyte count < 1500 / mm3, and C-reactive protein > 5 mg/l [20, 26–28]. A biochemical score was developed, considering the presence of at least three altered biochemical factors as strongly suggestive of malnutrition. The MNA score was also used to evaluate the risk for malnutrition.

Finally, the study explored the predictive capabilities of these scores for sarcopenia, both individually and in combination, within cirrhotic and non-cirrhotic subpopulations. Baseline characteristics of study participants were evaluated based on liver status (non-cirrhotic vs. cirrhotic) and sarcopenia status (non-sarcopenic vs. sarcopenic) using appropriate statistical tests, including chi-square tests for categorical variables, *t* tests for continuous variables and the Mann–Whitney U test for non-parametric variables. Pearson correlation coefficients were calculated to assess the relationship between estimated muscle mass and laboratory-anthropometric parameters in the general population and its subclasses of cirrhotic and non-cirrhotic patients. A logistic regression model was applied to evaluate the association between sarcopenia and the classes of biochemical score, MNA score, and their combination. Odds ratios (OR) and 95% confidence intervals (CI) were calculated to quantify these associations. To assess the discriminative ability of each prognostic score, ROC curves and corresponding areas under the curves (AUROCs) were calculated for the general population and the subgroups of non-cirrhotic and cirrhotic patients. Statistical analyses were conducted using Stata SE 15.0 (StataCorp, College Station, TX), and results were considered significant at a *p* value < 0.05 (95% confidence interval). This comprehensive and multifaceted approach ensured a thorough examination of sarcopenia and its association with various factors in the elderly population with advanced chronic liver disease.

## Results

From the cohort of elderly subjects admitted to the Hepatology and Geriatrics Unit, we recruited 115 patients, subsequently divided into two groups: cirrhotic n = 50 (43.47%) and non-cirrhotic n = 65 (56.53%). The overall population was predominantly female (53%), with the most common comorbidity being arterial hypertension (84.3%), followed by type 2 diabetes mellitus (43.47%) and dyslipidemia (43.47%). The analyzed patient cohort had a mean age of 78.4 ± 8.1 years, and the average BMI was 28.30 ± 6.43 kg/m2 (overweight), with a mean MNA score indicative of a risk of malnutrition (22.17 ± 3.62) (Table 1). The mean ASM calculated according to Hwang's equation was 19.31 ± 4.05 kg. In addition, upon admission, the population presented with mild anemia (mean Hb 10.91 ± 2.29 g/dl), elevated serum GGT levels (101.25 ± 153.92 U/L), mild renal insufficiency (mean creatinine 1.28 ± 1.03 mg/dl), altered coagulative status with an INR of (1.33 ± 0.45), and increased inflammatory indices (ESR 54.89 ± 28.35 mm/h and CRP 37.35 ± 53.51 mg/dl) (data not shown). The study population was stratified according to the presence/absence of cirrhosis. Differences between cirrhotic and non-cirrhotic patients regarding anthropometric data were analyzed, revealing no significant differences in the two populations. From the analysis of laboratory data, the two populations (patients with advanced chronic liver disease and non-cirrhotic patients) appeared homogeneous for parameters such as hemoglobin, lymphocyte count, ALT, GGT, creatinine, albumin, transferrin, total cholesterol, HDL cholesterol, LDL cholesterol, triglycerides, ESR, CRP, and HbA1c. However, cirrhotic patients exhibited reduced platelet and white blood cell counts compared to non-hepatopathic patients (p < 0.001), while total and indirect bilirubin, AST, ALP, and INR were significantly increased compared to their counterparts (Table 1).

**Table 1:** Clinical and laboratory data of all patients, providing a comprehensive overview of relevant variables for a thorough understanding of the study cohort

Patient characteristic	n (115)	%	
Male	54	46.95	
Female	61	53.05	
Cirrhosis	50	43.47	
Non-cirrhosis	65	56.53	
Coronary artery disease	32	27.8	
Arterial hypertension	97	84.3	
Dyslipidemia	50	43.47	
COPD	25	21.73	
Type 2 diabetes mellitus	50	43.47	
Biochemical and anthropometric characteristic	Cirrhosis	Non-cirrhosis	*p*
	(Mean ± SD or Median; IQR)	(Mean ± SD or Median; IQR)	
Age (years)	78; 11,5	80; 11	0.34
Height (cm)	162,94 ± 8,73	162,78 ± 8,32	0.92
Weight (kg)	74,81 ± 12,66	74.94 ± 19,62	0.96
BMI (kg/m2)	28,26 ± 4.89	28,18 ± 7,40	0.94
Mid-arm circumference (cm)	26,1 ± 4,56	27,26 ± 5,28	0.20
Calf circumference (cm)	33,69 ± 5,84	33,76 ± 6,19	0.95
Handgrip (kg)	19,67 ± 8,95	19,49 ± 9,67	0.91
MNA (points)	22,5; 5,125	22,75; 5,125	0.87
Hemoglobin (g/dl)	11,00 ± 1,99	10,84 ± 2,51	0.69
Platelet (× 103 /ul)	132,34 ± 110,9	242,95 ± 98,2	< 0.001
White blood cells (× 103 /ul)	6,18 ± 2,90	9,27 ± 4,54	< 0.001
Lymphocytes (× 103 /ul)	1,40 ± 0,82	1,41 ± 0,68	0.94
Total bilirubin (mg/dl)	0,58; 0,38	0,3; 0,38	0.001
Indirect bilirubin (mg/dl)	0,875; 0,38	0,4; 0,38	0.002
GOT (UI/L)	35,5; 12	19; 12	0.009
GPT (UI/L)	24,5; 13	17; 13	0.13
GGT (U/L)	61,5; 78,5	24,5; 78,5	0.51
ALP (U/L)	128,5; 42	75; 42	0.03
Creatinine (mg/dl)	1,02; 0,79	1,12; 0,79	0.20
INR	1,32; 0,26	1,12; 0,26	0.01
Albumin (gr/dl)	3,06 ± 0,60	3,17 ± 0,48	0.29
Transferrin (mg/dl)	230,9 ± 83,93	231,2 ± 84,72	0.98
Total cholesterol (mg/dl)	136,36 ± 47,28	138,87 ± 44,23	0.77
HDL cholesterol (mg/dl)	32,70 ± 14,29	36,62 ± 13,92	0.15
LDL cholesterol (mg/dl)	84,10 ± 41,14	84,67 ± 35,64	0.93
Triglyceride (mg/dl)	81,5; 36	98; 36	0.09
ESR(mm/h)	54; 38	54,5; 38	0.79
CRP (mg/l)	12; 52,875	13,2; 52,875	0.27
HbA1c (%)	5,9; 1,2	6,1; 1,2	0.37

MNA, Mini Nutritional Assessment; GOT, Glutamic Oxaloacetic Transaminase; GPT, glutamine-pyruvate transaminase; GGT, gamma-glutamyl transpeptidase; ALP, alkaline phosphatase; INR, international normalized ratio; HDL, high-density lipoprotein; LDL, low-density lipoprotein; ESR, Erythrocyte sedimentation rate; CRP, C-reactive protein; HbA1c, glycated hemoglobin

Applying the criteria set forth by the European Working Group on Sarcopenia in Older People, the patient cohort was subdivided into those classified as normal and those deemed to have probable sarcopenia. In addition, individuals with probable sarcopenia were further categorized: if they exhibited deficits in both muscle mass and strength, they were classified as Confirmed Sarcopenia; however, if their loss was limited to strength alone, they were categorized as dynapenic (Table 2).

**Table 2:** Distribution of sarcopenia within the study population

	Cirrhosis (n 50) %	Non Cirrhosis (n 65) %	*p*
Normal	38%	36.92%	n.s
Probable Sarcopenia	62%	63.07%	n.s
Confirmed Sarcopenia	14%	18.46%	n.s
Dynapenia	48%	44.61%	n.s

Significant correlations were observed between Appendicular Skeletal Muscle Mass (ASM), calculated using Hwang's formula, and various anthropometric and laboratory parameters in the general population. Positive correlations were found for height (*r* = 0.73; *p* < 0.001), weight (*r* = 0.67; *p* < 0.001), BMI (*r* = 0.33; *p* < 0.001), Mid-arm circumference (*r* = 0.29; *p* < 0.001), Calf circumference (*r* = 0.56; *p* < 0.001), handgrip (*r* = 0.58; *p* < 0.001), and MNA (*r* = 0.33; *p* < 0.001). In addition, ASM exhibited inverse correlations with total cholesterol (*r* = -0.25; *p* = 0.005), HDL cholesterol (*r* = -0.2; *p* < 0.001), LDL cholesterol (*r* = -0.2; *p* = 0.01), and ESR (*r* = -0.18; *p* = 0.04).

Furthermore, we deepened the correlation between ASM and anthropometric and laboratory parameters in the subpopulation of cirrhotic and non-hepatopathic patients. Significant correlations were found for the parameters indicated below (Table 3).

**Table 3:** correlation between ASM and anthropometric and laboratory parameters in cirrhotic and non- cirrhotic patients. Abbreviations: BMI, body mass index; MNA, mini nutrional assessment, GPT, glutamine-pyruvate transaminase; LDL, low-density lipoprotein

	Cirrhosis		Non Cirrhosis	
	*r*	*p*	*r*	*p*
Height (cm)	0,83	< 0.001	0,68	< 0.001
Weight (kg)	0,55	< 0.001	0,73	< 0.001
BMI (kg/m^2^)	0,02	0.86	0,47	< 0.001
Mid-arm circumference (cm)	0,12	0.38	0,39	< 0.001
Calf circumference (cm)	0,46	< 0.001	0,62	< 0.001
Handgrip (kg)	0,53	< 0.001	0,61	< 0.001
MNA (point)	0,21	0.13	0,41	< 0.005
GPT (U/L)	-0,27	< 0,04	0,11	0.37
Total cholesterol (mg/dl)	-0,06	0.64	-0,39	< 0.001
Cholesterol LDL (mg/dl)	-0,03	0.83	-0,43	< 0.001

The ability of laboratory parameters (biochemical score), MNA (MNA score), and their combination (Biochemical Score and MNA) to predict the probability of sarcopenia in the general population was assessed by determining the odds ratio (OR): notably, the biochemical score is unable to predict the risk of developing sarcopenia in the general population. MNA proves to be a strong predictor, reaching statistical significance (OR: 4,54, *p* < 0,02). The combined use of the biochemical score and MNA allows for the highest odds ratio with statistical significance (OR: 8, *p* < 0,01). (Table 4).

**Table 4:** odds ratios (OR), confidence intervals (C.I.), sample sizes (N), and corresponding p-values for the assessment of sarcopenia risk using different predictors in the general population and in cirrhotic and non- cirrhotic patients

	OR TOT	C.I	*N*	*p*		
Biochemical Score	1.98	0.53–7.36	115	0.307		
MNA Score	4.54	1.26–16.30	115	0.020		
Biochemical Score and MNA	8	1.42–44.91	37	0.018		
	Non Cirrhosis (OR)	N	C.I	Cirrhosis (OR)		C.I
Biochemical Score	0.53	65	0.41- 0.66	0.57	N	0.42–0.73
MNA Score	0.58	65	0.45- 0.72	0.60	50	0.42–0.79
Biochemical Score and MNA	0.72	18	0.46- 0.98	0.73	–	0.39–1.00

**Fig. 1:**
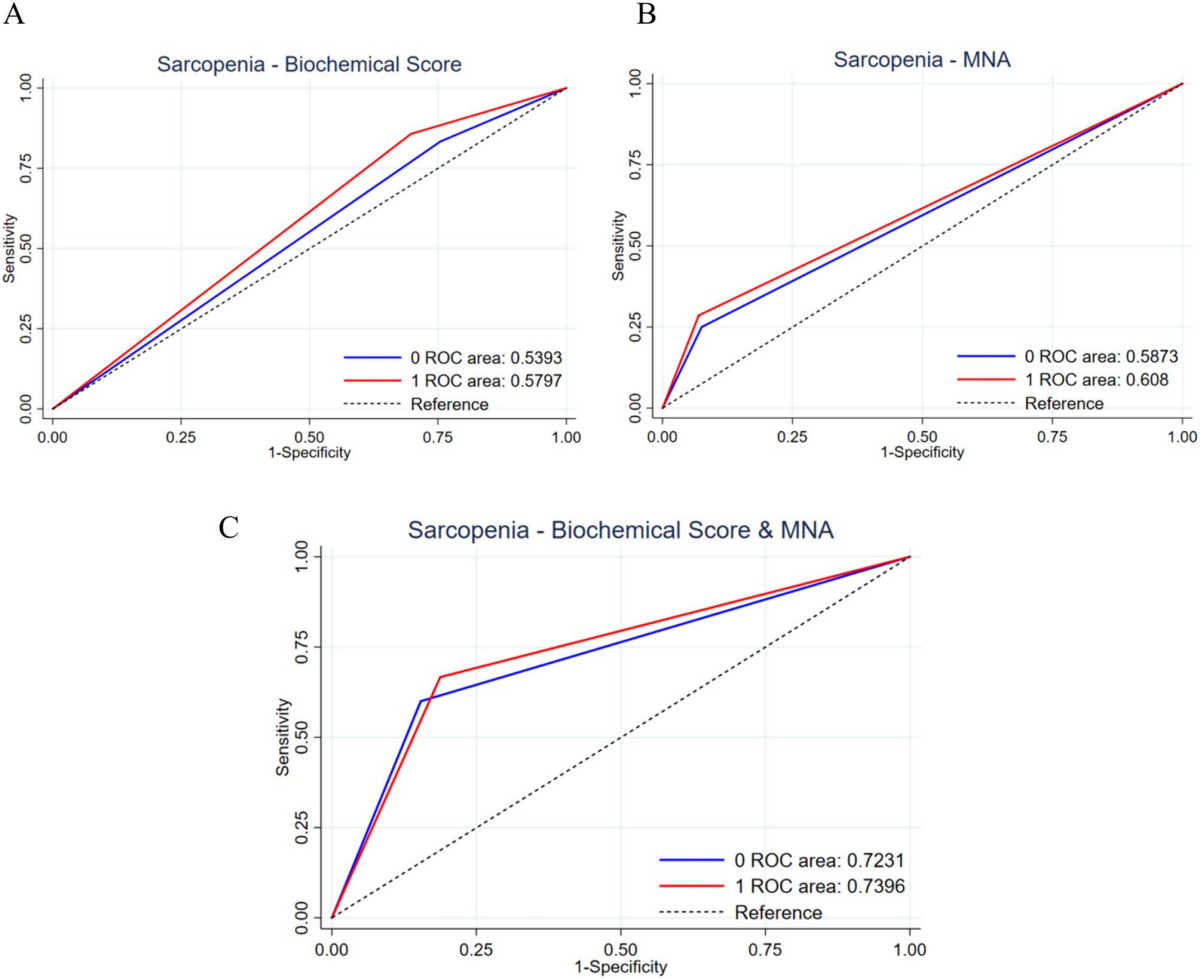
Receiver Operating Characteristic (ROC) curves depict the predictive performance for Sarcopenia in cirrhotic (red line) and non-cirrhotic patients (blue line). Using the Biochemical Score (A), the Area Under the ROC Curve (AUROC) is 0.53 for non-cirrhotic and 0.57 for cirrhotic patients. For MNA (B), the AUROC is 0.58 for non-cirrhotic and 0.60 for cirrhotic patients. The combined use of Biochemical Score and MNA (C) yields AUROC values of 0.72 for non-cirrhotic and 0.73 for cirrhotic patients

Therefore, we generated the ROC curve to explore such statistical model in predicting sarcopenia in the main subpopulations: cirrhotic and non-hepatopathic patients (Fig. 1).

## Discussion

Our study aims to analyze the impact of sarcopenia in hospitalized elderly patients. We deliberately conducted the study in a cohort of cirrhotic patients, comparing it with non-liver disease patients to highlight differences. Thus, the elderly cirrhotic population often develops sarcopenia. Therefore, we aimed to verify whether the characteristics of sarcopenia in this population were consistent with those of the general reference population. The explored population exhibited significant comorbidities, including arterial hypertension, type 2 diabetes mellitus, and dyslipidemia. Clinical features included increased BMI and a risk of malnutrition (assessed by MNA). The population was stratified into elderly patients with advanced liver disease and compared with those without such pathology. Cirrhotic patients accounted for 43.47% of the total population. Both cohorts, in terms of anthropometric assessment, muscle strength (analyzed by handgrip), and nutritional evaluation, were homogeneous. Laboratory parameters differed between cirrhotic and non-liver disease patients, with cirrhotic patients showing reduced platelet and white blood cell counts and elevated total and indirect bilirubin, GOT, ALP, and INR. These conditions are attributed to underlying pathology, including portal hypertension-associated splenomegaly and hepatic synthesis deficit. Patients were classified based on the European Working Group on Sarcopenia in Older People. Probable sarcopenia was identified with a prevalence of 62% in cirrhotic patients and 63% in non-cirrhotic patients, with no statistically significant differences observed. As known, the original operational definition of sarcopenia by EWGSOP was a major change at that time, as it added muscle function to former definitions based only on detection of low muscle mass. Revised guidelines, consider muscle strength the forefront, as it is recognized that strength is better than mass in predicting adverse outcomes [23]. Based on the loss of muscle strength, probable sarcopenic patients were further classified into two groups: “confirmed sarcopenia” (those who experienced both muscle mass and strength loss) and “dynapenic” (those who only lost muscle strength). We did not find any significant difference in the prevalence of this condition between cirrhotic and non-cirrhotic patients. However, considering the hypoalbuminemia typically encountered in cirrhosis, we would have expected some significant differences regarding dynapenia. This lack of distinction may be attributed to the small number of individuals in the subpopulation.

To better define whether there is a relationship between muscle mass and malnutrition, we performed a correlation analysis between appendicular muscle mass and the previously listed anthropometric-laboratory parameters. Positive correlations were observed between appendicular mass and height, weight, BMI, arm circumference, calf circumference, handgrip, and MNA, indicating that ASM assessment correlates with anthropometric parameters, malnutrition risk, and grip strength. An inverse correlation was found between ASM and total cholesterol, HDL, LDL, and ESR, suggesting a relationship between muscle mass and lipid profile and inflammation. Literature supports the concept of “lipotoxicity,” a pathologic condition explaining the evolution process in sarcopenia, indicating a vicious cycle between sarcopenia and ectopic fat accumulation through mitochondrial dysfunction, proinflammatory cytokine production, oxidative stress, collagen deposition, extracellular matrix remodeling, and lifestyle habits. The exacerbation of lipotoxicity in sarcopenia may lead to increased disability, morbidity, and mortality [29, 30]. The correlation between ASM and collected parameters was further explored in cirrhotic and non-liver disease patients. Both populations showed positive correlations for height, weight, calf circumference, and handgrip. In non-liver disease patients, ASM also correlated positively with BMI, MNA, and arm circumference but negatively with total cholesterol and LDL, suggesting the influence of malnutrition degree and lipotoxicity on sarcopenia in non-liver disease patients. In contrast, in cirrhotic patients, appendicular mass correlated negatively with GPT values, a liver damage-specific index, indicating that cirrhotic patients with greater liver damage had lower appendicular muscle mass. It's worth noting that cirrhotic patients showed no correlation with the MNA, suggesting that sarcopenia in cirrhosis is not correlated with malnutrition but rather with the alteration of the muscular component. The presence of sarcopenia has been shown to be associated with increased risk of falls and fractures, development of acute decompensation or acute-on-chronic liver failure, and death in patients with cirrhosis [31]. In a recent extensive meta-analysis, Tantai et al. illustrated that sarcopenia affects approximately one-third of patients with cirrhosis, correlating with a roughly twofold higher risk of death among affected patients. This association remained consistent across nearly all patient subgroups, including those with low MELD scores [2].

Therefore, we questioned how we could stratify the prognosis of cirrhotic and sarcopenic patients quickly and non-invasively. Analysis of the odds ratio in the elderly hospitalized population for parameters associated with sarcopenia demonstrated a strong capability to assess the likelihood of sarcopenia, especially when utilizing the MNA score in conjunction with the biochemical score. Finally, the analysis of AUROC, evaluating the test's accuracy in predicting sarcopenia, demonstrated good sensitivity when employing the composite score incorporating altered nutritional risk markers and reduced MNA. These results are consistent with literature emphasizing the importance of malnutrition in sarcopenia assessment, associated with about four times higher risk of developing it, sometimes in a severe form [32, 33].

In conclusion, considering the progressive demographic aging, sarcopenia will have a strongly negative prognostic and economic impact on public health. It is associated with an increased risk of short- and long-term mortality, reduced overall survival, an increased risk of serious complications, and prolonged hospitalization [34]. Our study demonstrated that sarcopenia is a pathologic condition present in the hospital setting, both in the general population and in patients with liver cirrhosis, with no significant differences in terms of prevalence. Sarcopenic patients exhibited lower anthropometric and malnutrition screening test values than their non-sarcopenic counterparts. Calculated muscle mass in the cohort of hospitalized elderly patients correlated directly with anthropometric parameters, especially with MNA, and thus with the degree of malnutrition, while being inversely correlated with lipid profile and systemic inflammation. When analyzing the association between ASM in cirrhotic and non-liver disease patients, significant differences emerged, such as the direct correlation in non-cirrhotic patients with the MNA value compared to cirrhotic patients, and the inverse correlation with the lipid profile present in the non-liver disease population compared to cirrhotic patients. Both populations, however, showed a direct correlation with height, weight, calf circumference, and handgrip, emphasizing the link between muscle strength and muscle mass. Finally, in cirrhotic patients, muscle mass was inversely correlated with GPT, indicating the role of chronic liver damage.

The main limitation of the study is the small number of individuals in the subpopulation, particularly when considering the distinction between confirmed sarcopenia and dynapenia. This limitation hinders the ability to draw significant conclusions regarding the expected differences, especially regarding dynapenia, in the context of hypoalbuminemia typically encountered in cirrhosis. In addition, the study acknowledges the potential influence of the small sample size on the observed relationships and correlations, which may limit the generalizability of the findings. To address this limitation, forthcoming studies could focus on enrolling larger cohorts of patients, enabling more thorough analyses and comparisons across subgroups. Furthermore, longitudinal studies could be undertaken to evaluate the progression of sarcopenia over time and its effects on clinical outcomes in elderly patients with liver disease.

Our analyses revealed that assessing nutritional status using MNA and combining it with biochemical markers of nutritional deficiency, such us transferrin, albumin, total cholesterol, lymphocyte count and C-reactive protein is more sensitive and specific for screening sarcopenia in hospitalized elderly patients, even in the absence of significant differences between cirrhotic and non-cirrhotic patients. In our study, we initially recognized the potential value of incorporating validated biochemical scores such as the CONUT score or Prognostic Nutritional Index (PNI) to assess nutritional status and predict adverse outcomes in in-hospital patients across diverse settings [35, 36]. However, upon closer examination, we encountered limitations in the ability of these scores to adequately define the state of sarcopenia in cirrhotic patients, particularly those in end-stage disease. The inclusion of total cholesterol count in the CONUT score and the reliance on lymphocyte count in both the CONUT and PNI scores posed challenges in accurately characterizing sarcopenia in this specific patient population. Consequently, we chose to augment our analysis by integrating nutritional parameters with anthropometric and biochemical indicators such as transferrin and C-reactive protein. This approach allowed for a more comprehensive evaluation of sarcopenia in cirrhotic patients, with particular attention to its impact on muscle mass. Furthermore, we employed the Hwang equation to estimate appendicular muscle mass, enhancing the precision of our assessments.

Collectively, our findings indicate that: (1) sarcopenia should be included in the initial assessment of all patients with cirrhosis; (2) regular monitoring for sarcopenia is warranted in all cirrhosis patients, irrespective of the severity of hepatic dysfunction; (3) further research is essential to integrate sarcopenia or muscle mass index/function into a structured prognostic scale for cirrhosis patients.

Our study achieved its primary endpoint by assessing sarcopenia in elderly hospitalized patients with advanced chronic liver disease and comparing it with non-hepatopathic patients, while examining correlations with anthropometric and biochemical factors. In addition, the secondary endpoint was achieved by identifying a diagnostic test capable of predicting the risk of sarcopenia in the elderly, particularly in cirrhotic patients at high risk of malnutrition. Therefore, a thorough screening and assessment of sarcopenia and nutritional status in hospitalized patients should be carried out. This is crucial for implementing nutritional and multidisciplinary intervention programs, especially in liver-suffering populations at high risk of disease and loss of independence.

## Data Availability

The participants of this study did not give written consent for their data to be shared publicly, so due to the sensitive nature of the research supporting data is not available.
